# The amblyopic eye in subjects with anisometropia show increased saccadic latency in the delayed saccade task

**DOI:** 10.3389/fnint.2014.00077

**Published:** 2014-10-14

**Authors:** Maciej Perdziak, Dagmara Witkowska, Wojciech Gryncewicz, Anna Przekoracka-Krawczyk, Jan Ober

**Affiliations:** ^1^Laboratory for Oculomotor Research, Department for Biophysical Measurements and Imaging, Nałęcz Institute of Biocybernetics and Biomedical Engineering, Polish Academy of SciencesWarsaw, Poland; ^2^Laboratory of Vision Science and Optometry, Faculty of Physics, Adam Mickiewicz University of PoznanPoznan, Poland

**Keywords:** amblyopia, anisometropia, saccadic latency, anisometropic amblyopia, delayed saccade, saccadometry

## Abstract

The term *amblyopia* is used to describe reduced visual function in one eye (or both eyes, though not so often) which cannot be fully improved by refractive correction and explained by the organic cause observed during regular eye examination. Amblyopia is associated with abnormal visual experience (e.g., anisometropia) during infancy or early childhood. Several studies have shown prolongation of saccadic latency time in amblyopic eye. In our opinion, study of saccadic latency in the context of central vision deficits assessment, should be based on central retina stimulation. For this reason, we proposed saccade delayed task. It requires inhibitory processing for maintaining fixation on the central target until it disappears—what constitutes the GO signal for saccade. The experiment consisted of 100 trials for each eye and was performed under two viewing conditions: monocular amblyopic/non-dominant eye and monocular dominant eye. We examined saccadic latency in 16 subjects (mean age 30 ± 11 years) with anisometropic amblyopia (two subjects had also microtropia) and in 17 control subjects (mean age 28 ± 8 years). Participants were instructed to look at central (fixation) target and when it disappears, to make the saccade toward the periphery (10°) as fast as possible, either left or the right target. The study results have proved the significant difference in saccadic latency between the amblyopic (mean 262 ± 48 ms) and dominant (mean 237 ± 45 ms) eye, in anisometropic group. In the control group, the saccadic latency for dominant (mean 226 ± 32 ms) and non-dominant (mean 230 ± 29 ms) eye was not significantly different. By the use of LATER (Linear Approach to the Threshold with Ergodic Rate) decision model we interpret our findings as a decrease in accumulation of visual information acquired by means of central retina in subjects with anisometropic amblyopia.

## Introduction

The term *amblyopia* is used to describe reduced visual function in one eye (or both eyes, though not so often) which cannot be fully improved by refractive correction and explained by the organic cause observed during regular eye examination (Barrett et al., 2004). However, amblyopia is reversible (especially when detected in early childhood) in most cases by proper therapy. This is usually a unilateral developmental disorder of spatial vision which affects about 2–5% of the population and is associated with abnormal visual experience during infancy or early childhood (Roper-Hall, 2007). The most common risk factors for developing amblyopia are anisometropia (unequal refractive error in the two eyes), strabismus (misalignment of visual axes) and another form of deprivation (e.g., congenital cataract) early in life (Kiorpes and McKee, 1999; Roper-Hall, 2007). In the case of anisometropic amblyopia, peripheral retina is generally normal (Yu et al., 1998; Pardhan and Whitaker, 2000), and spatial vision deficits are the consequence of chronic blur in the area of central retina during sensitive period for development of visual acuity (see Daw, 1998). Anisometropia becomes clinically significant when its magnitude reaches approximately 1 D in either one or both meridians (Benjamin, 2006). It is generally accepted that strabismic and anisometropic amblyopia are associated with an active inhibition of visual input originating in the fovea of the deviating and more ametropic eye respectively (Von Noorden and Campos, 2002). In practice, amblyopia is recognized on the basis of reduced visual acuity on Snellen chart, despite the optimal refractive correction and a period of refractive adaptation (Stewart et al., 2004). Apart from evident deficits in optotype acuity, subjects with amblyopia also manifest deficits in other visual functions including reduced contrast sensitivity (Bradley and Freemen, 1981; McKee et al., 2003), spatial and temporal crowding (Bonneh et al., 2007), reduced grating and Vernier acuity (McKee et al., 2003), spatial uncertainty (Demanins and Hess, 1996), temporal instability and spatial distortions (Sireteanu et al., 2008) or prolongation of visual reaction time for amblyopic eye (Hamasaki and Flynn, 1981; Nuzzi et al., 2007). Increased latency of the evoked potentials was also reported in amblyopia (Sokol, 1983; Parisi et al., 2010) but features such as critical flicker frequency and color vision remains generally normal (Roper-Hall, 2007). Moreover, subjects with amblyopia often suffer lack of (or reduced) binocular function, especially stereopsis (Roper-Hall, 2007).

**Figure 1:**
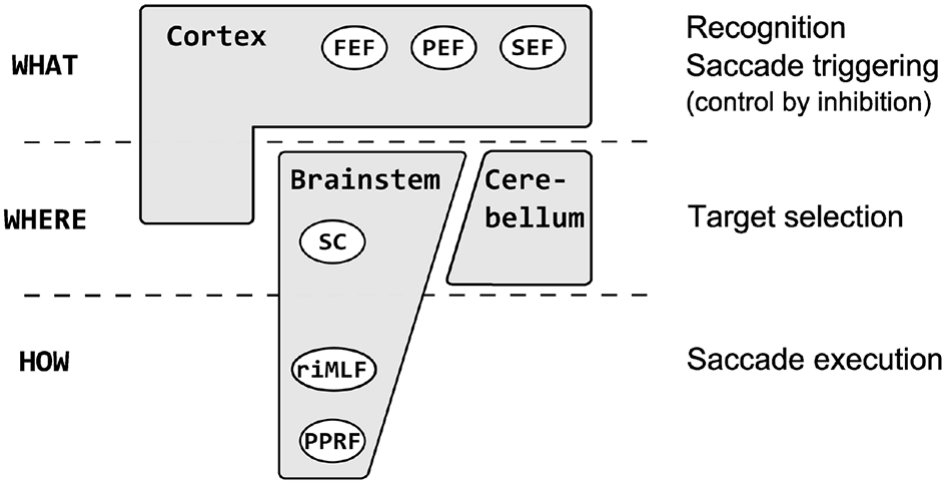
**The saccadic system hierarchy**. At the lowest level are horizontal gaze centers—burst neurons in the paramedian pontine reticular formation (PPRF) and vertical gaze centers—burst neurons in the midbrain rostral interstitial nucleus of the medial longitudinal fasciculus (riMLF) which generate saccadic command to the extraocular muscles. Above the PPRF and riMLF level are the superior culliculus (SC), important for target selection and initiation of eye movement (supplemented also by cerebellum and the cortex). At the top of this hierarchy are the cortical areas: the frontal eye field (FEF), the parietal eye field (PEF) and the supplementary eye field (SEF) involved in recognition, inhibition, and decision about the movement.

Quite a high number of neuroimaging (see Anderson and Swettenham, 2006) and psychophysical (see Von Noorden and Campos, 2002) studies were conducted in amblyopic subjects but surprisingly only few of them were focused on oculomotor behavior (especially saccades). Saccades are fast (up to ~500°/s) and brief (typically ~30–100 ms) eye movements which redirect the fovea between successive points of visual scene (Leigh and Zee, 2006; Munoz et al., 2007). The central retina (more precisely *fovea centralis* with the central pit called *foveola* ~0.35 mm across and ~1.2° of the visual field) is physiologically predestined for acquisition of the visual image with high resolution and greatest visual acuity (Moses and Hart, 1987; Munoz et al., 2007). Apart from obvious gaze shifting function, human oculomotor system also ensures the stable retinal image and prevents visual fading. Clear and stable retinal image is needed for proper visual development and perception. Saccades are generated in the brainstem and triggered at the level of cerebral hemispheres (Pierrot-Deseilligny et al., 2002). There is a specific three–level hierarchy in oculomotor system, that can be summarized as *what* (recognition), *where* (localization) and *how* (pattern of neuronal excitation required for execution) (Carpenter, 2004a) (Figure 1). At the lowest level of this hierarchy are horizontal and vertical gaze centers, respectively the burst neurons in the paramedian pontine reticular formation (PPRF) and the burst neurons in the midbrain rostral interstitial nucleus of the medial longitudinal fasciculus (riMLF) (Leigh and Zee, 2006). They send saccadic command to the motoneurons innervating horizontally and vertically acting extraocular muscles (Leigh and Zee, 2006). Above the PPRF and riMLF level are the superior colliculus (SC)—area important for target selection and initiation of eye movement (this task is supplemented also by the cerebellum and the cortex) (Carpenter, 2004a; Leigh and Zee, 2006). At the top of this hierarchy are the cortical areas involved in saccade control by inhibition of unnecessary collicular mechanisms. They can be thought of as preventing the collicular route from operating, so the saccadic reaction times are longer than they might otherwise be (Carpenter, 2004a). Experimental studies (Schall and Hanes, 1993; Gaymard et al., 1998; Johnston and Everling, 2008) have shown that three cerebral regions are involved in triggering saccades: the frontal eye field (FEF), the parietal eye field (PEF) and the supplementary eye field (SEF). The FEF is mainly involved in the control of voluntary saccades (Vernet et al., 2014) (e.g., intentional—visually guided saccades, antisaccades, delayed saccades, memory guided saccades). The SEF appears crucial in learning phase (presentation of the visual target) (Hardman Lea et al., 1991; Müri et al., 1995) and just after the GO signal for saccade (Müri et al., 1994). The PEF is believed to be involved in the triggering of reflexive, visually guided saccades (Hanes and Schall, 1996; Pierrot-Deseilligny et al., 2002). One of the most interesting parameters of saccadic eye movement is the saccadic reaction time (latency). The interval between stimuli presentation and the beginning of eye movement is typically about 200–250 ms and is surprisingly variable (Hanes and Schall, 1996; Carpenter, 1999, 2004b; Liversedge et al., 2011) Saccadic latency is also much longer than it would be expected from synaptic delays and nerve conduction (the retino–collicular route takes about 60 ms) (Carpenter, 1999) because of higher, cortical levels involved in decisions about whether to respond to a stimulus or not (Carpenter, 1988, 1999; Reddi and Carpenter, 2000).

**Figure 2:**
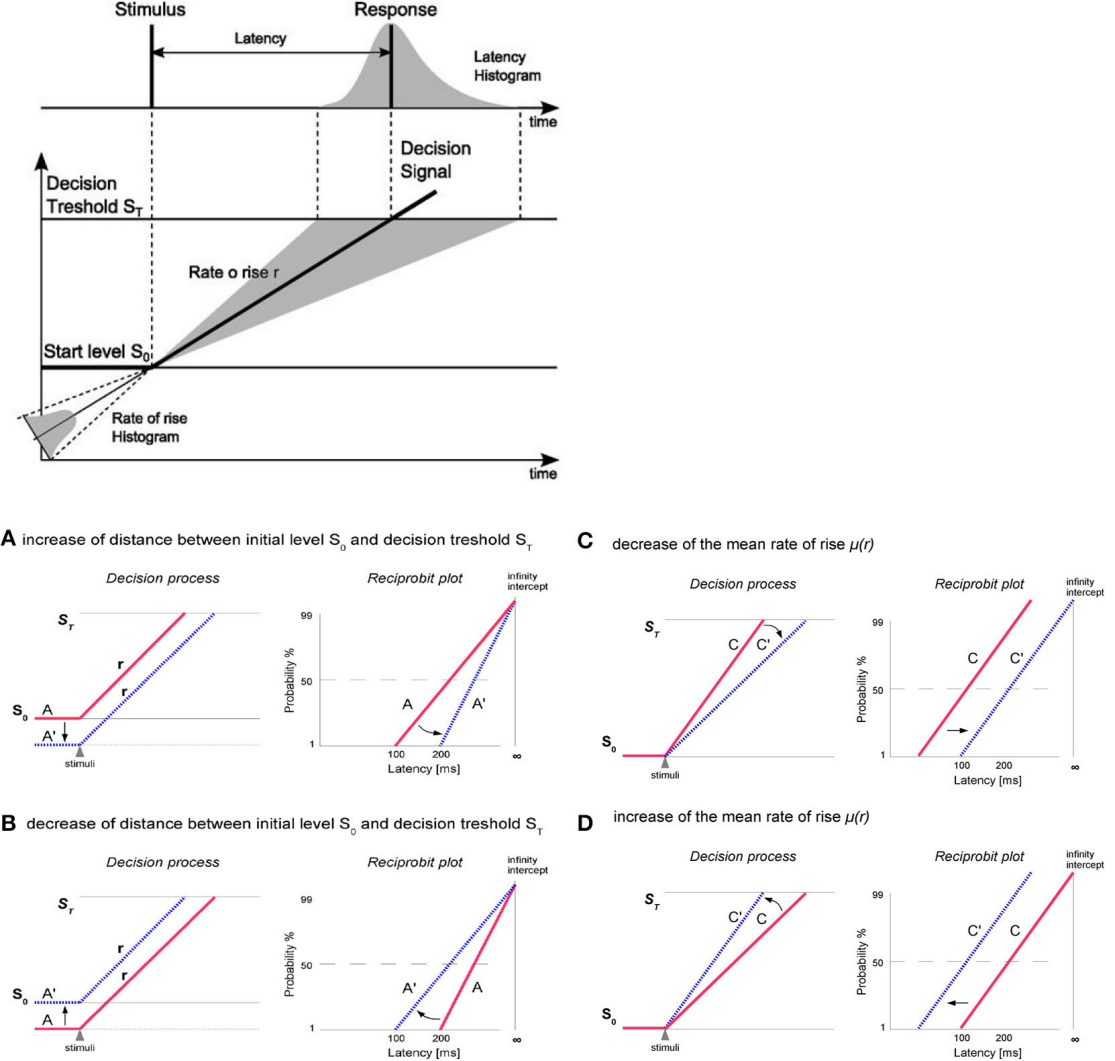
**At the top:** The LATER model. At the moment of target presentation, the decision signal starts form initial level S_O_ and rises linearly with a rate *r* until reaching the decision threshold S_T_ what causes the generation of saccade. The rate of rise varies randomly from trial to trial (about a mean μ), resulting in a skewed distribution toward the responses with longer latencies. **At the bottom:** Changes in the parameters of the LATER model have distinct effects on the reciprobit plot. **(A,B)** Alternations in the distance between S_T_ and S_O_ (here evoked by So change, but S_T_ may also by modified) causes swiveling of the distribution line about the point (I) where it intersects the vertical infinite-time axis. Parameter S_T_ can be modified due to a different instruction given to the subjects (e.g., “react as fast as possible” or “react as accurate as possible”). Variation in S_0_ can be caused by a different probability of the target dislocation. **(C,D)** Changes in the rate of information supply affect the mean (μ) rate of rise of the decision signal and causes parallel shift of the distribution line along the time axis (leftward-reduction of latency or rightward-increase of latency) without a change in the slope.

Generation of saccades is closely connected with decision making processes (reflected by the saccadic latency). Carpenter's model LATER (Linear Approach to Threshold with Ergodic Rate) suggests that at the moment of target presentation, the decision signal starts from initial level S_0_ and rises linearly with a rate (r) until it reaches the decision threshold S_T_ which causes the generation of saccade (Figure 2). The rate of rise (r) varies randomly from trial to trial (about a mean μ and with variance σ^2^) and its variability exhibits characteristics of a normal distribution. According to the model LATER the level S_0_ represents the logarithm of the prior probability whilst the threshold S_T_ reflects the urgency of reaction. The μ (mean of the rate of rise of the decision signal as well as the mean reciprocal latency) (Noorani, 2014) can be treated as the supply of information (Carpenter, 1981; Reddi and Carpenter, 2000) whereas the σ reflects the variability in the latency distributions (Carpenter and Williams, 1995; Noorani, 2014). Saccadic latency, is characterized by a skewness of distribution toward the greater values (Reddi and Carpenter, 2000). Although the variability of saccadic reaction time (latency) is large, in general, under high-contrast conditions, it follows a simple rule: the reciprocal of latency obeys a Gaussian (*recinormal*) distribution (Carpenter, 1988). Since latency and rate (r) are reciprocally related, Carpenter (1981) proposed to analyze not the distribution of latency (T) but the distribution of its reciprocal 1/T (*promptness*). When we plot the latency distribution as a function of its promptness, we obtain a curve that is very close to normal distribution (Carpenter, 1988). Using a probit scale, we can transform the cumulative Gaussian distribution to a straight line (a *reciprobit plot*, where the *x* axis has a reciprocal scale and the *y* axis has a probit scale) whose intercept with the 50% line represents the median and whose slope is directly related to standard deviation (SD) (Reddi et al., 2003; Coubard, 2012). The intercept represents the probability of not making a saccade at all (Carpenter, 1981; Reddi and Carpenter, 2000). Changes in the parameters of the LATER model have distinct effects on the reciprobit plot (Figure 2). Alternations in the distance between S_T_ and S_O_ causes swiveling of the distribution line about the point (I) where it intersects the vertical infinite-time axis. Parameter S_T_ can be modified due to a different instruction given to the subjects (“react as fast as possible” or “react as accurate as possible”). Variation in S_0_ can be caused by a different probability of the target dislocations (Carpenter, 1981, 1999; Reddi and Carpenter, 2000; Reddi et al., 2003). Changes in average rate of rise (μ) shifts the plot parallel along the time axis (leftward-reduction of latency or rightward-increase of latency) without a change in the slope (Carpenter, 1999; Reddi and Carpenter, 2000; Reddi et al., 2003; Coubard, 2012). The parallel shift of reciprobit plot reflects changes in the rate of information supply to the visual system (Reddi and Carpenter, 2000; Coubard, 2012).

Only few studies have dealt with saccadic latency in amblyopia and most of them have shown the increased latency during viewing with the amblyopic eye (see Mackensen, 1958; Ciuffreda et al., 1978; Niechwiej-Szwedo et al., 2010). However, previous studies of saccadic latency in amblyopia were focused on reflexive saccades, which are initiated in response to novel exogenous stimuli (Johnson et al., 2012), and neither of them have examined more complex saccadic responses (e.g., delayed saccades), which additionally involve the frontal cortex (FEF) and enable the central (visually deprived) retina to be engaged in a higher degree in the programming and saccade execution process. Reflexive saccades to the location of novel target depend primarily upon the direct projections from the occipital (visual) and parietal cortices to the superior colliculus (LeVasseur et al., 2001; McDowell et al., 2008). More complex, volitional saccades (e.g., delayed saccades) require additional neural regions to support the higher level processes (e.g., inhibition) and depend more upon the frontal cortex and its direct or indirect (via basal ganglia) projections to the superior colliculus (LeVasseur et al., 2001; McDowell et al., 2008).

In our opinion, the study of saccadic latency in the context of central vision deficits assessment, should be based on central retina stimulation. For this reason, we decided to study more complex saccadic responses during saccade delayed task. Proposed saccadic paradigm, requires inhibitory processing for maintaining fixation on the central target until it disappears, what constitutes the GO signal for saccade (Pierrot-Deseilligny et al., 2002; Munoz et al., 2007). In order to execute this kind of saccade, several processes are believed to occur: computation of the parameters of the movement, inhibition of the already prepared saccade, disengagement of visual attention from fixation position and finally decision to move (Coubard et al., 2003). In proposed saccadic paradigm, we may distinguish two phases:

- Initial phase: subject maintains visual fixation on the central target and suppresses the reflexive saccade toward the peripheral target. Due to potential amblyopic disorientation, the peripheral target is not displayed simultaneously with the central fixation target (as it is usually arranged in this kind of paradigm). Instead of that, the peripheral target is displayed after a delay long enough to signal to the amblyopic eye which of the appearing targets is the one to which saccadic response should be destined.
- Preparatory phase: the presence of both central (fixation) and peripheral target, enables the oculomotor system to acquire parameters needed for programming the saccadic response. The neural command for saccade (its amplitude and direction) is ready and waits for permission to be executed. Permission is provided by the GO signal being the change of the stimuli status received by means of the central retina.

Taking all into consideration, the central retina has a dominant effect on observed visual deficits in subjects with anisometropic amblyopia (Yu et al., 1998; Pardhan and Whitaker, 2000), thus we expect that the loss of physiological function of foveal vision in these individuals may affect specifically saccades in terms of their spatial and temporal properties during saccade delayed task.

Our hypothesis is, that subjects with anisometropic amblyopia will reveal differences in the saccadic latency distribution (increased latency in amblyopic eye in comparison to dominant eye), predominantly by parallel (rightward) shifting of the reciprobit plot. According to the LATER model this should be interpreted as a difference in supply of information (decrease in accumulation of visual information what delays the saccade execution decision) between the amblyopic and dominant eye.

Under physiological conditions, saccadic latencies in both eyes are generally equal (Carpenter, 1988), so it is reasonable (especially in cases of unilateral amblyopia) to compare saccadic latency distribution between the eyes. Thus, the aim of the current study is to investigate saccadic latency (by the use of the delayed task) in subjects with anisometropic amblyopia.

## Methods

### Participants

Sixteen participants with anisometropic amblyopia (mean age 30 ± 11 years) and seventeen control subjects (mean age 28 ± 8 years) took part in the study. All subjects underwent standard optometric examination, including the measurement of visual acuity (Snellen chart – decimal notation), refractive error examination (static retinoscopy and subjective refraction), the binocular vision examination (cover test at distance, Worth 4 dot test at far and near, stereopsis-stereo Fly test, phoria measurement at distance – Maddox) and slit lamp (anterior segment) examination. Since anisometropia may be associated with microtropia (Hardman Lea et al., 1991), we performed also several additional tests (Bagolini test, 4 base-out prism test and fixation pattern – visuscopy) in order to diagnose the existence of microtropia, defined as small angle heterotropia of less than 5° associated with harmonious anomalous retinal correspondence (ARC) (Lang, 1974). In addition, in subjects with microtropia amblyopia, normal or near normal peripheral fusion, reduced or absent stereoacuity, eccentric fixation, foveal (central) suppression scotoma are often present (Houston et al., 1998; Von Noorden and Campos, 2002).

In our experimental group fourteen subjects were orthotropic and two (No. 7 and No. 8) had microtropia “with identity” (no manifest movement on cover test, the eccentric fixation point coinciding with the angle of ARC) (Houston et al., 1998) (see Table 1 for clinical characteristic of individuals with amblyopia). Anisometropic (experimental) group (AG) consisted of subjects with anisometropic amblyopia according to the following criteria:

– no manifest strabismus during cover test;
– reduced best corrected visual acuity (BCVA) at least two lines in the amblyopic eye;
– coexistent difference in refractive error between the two eyes, greater than 1 diopter (D) in either one or both meridians;

**Table 1:** **Clinical characteristic of individuals with anisometropic amblyopia**.

**Subject**	**Age (years)**	**Refractive Error**		**Distance Snellen visual acuity**		**Stereopsis (arcseconds)**	**Cover test (at distance)**	**Phoria/Tropia (prism dioptres)**	**Fixation**	**Suppression distance (Bagolini test)**
		***RE***	***LE***	***RE***	***LE***					
1	35	+2.50 / −2.00 × 8	+3.75 / −0.75 × 180	0.9	0.4	140	No manifest movement	1 exophoria	Central, unsteady	Central suppression
2	22	+3.25 / −0.75 × 165	+2.25	0.7	1.2	60	No manifest movement	2 esophoria	Central	No
3^#^	25	+3.00 / −0.25 × 105	+0.25 / −0.50 × 145	0.8	1.2	200	No manifest movement	3 esophoria	Central	No
4^#^	38	+5.00 / −2.00 × 135	+2.00 / −1.00 × 10	0.5	1.0	60	No manifest movement	Orthophoria	Central, unsteady	No
5	14	plano	+2.50 / −0.50 × 35	1.2	0.8	200	No manifest movement	Orthophoria	Central	No
6^#^	53	plano / −0.25 × 90	−9.50 / −0.50 × 65	0.9	0.2	no	No manifest movement	Orthophoria	Central, unsteady	Central suppression
7^#^	23	+1.00 / −0.50 × 40	+0.50/−3.75 × 70	1.2	0.6	140	No manifest movement	Microtropia	Eccen. 2° nasal	Central suppression
8	43	+4.50 / −0.50 × 90	+2.50 / −0.50 × 80	0.15	1.0	400	No manifest movement	Microtropia	Eccen. 3° nasal	Central suppression
9	30	+1.75 / −5.00 × 175	plano / −2.00 × 12	0.6	1.0	60	No manifest movement	3 esophoria	Central, unsteady	No
10	20	+2.50 / −4.00 × 5	+0.50 / −0.75 × 165	0.6	1.2	200	No manifest movement	4 esophoria	Central, unsteady	Central suppression
11^#^	43	+3.50 / −1.00 × 145	+4.25 / −4.00 × 15	1.0	0.6	100	No manifest movement	2 esophoria	Central, unsteady	Central suppression
12	16	+4.00 / −1.50 × 170	+0.75 / −0.25 × 170	0.7	1.2	400	No manifest movement	2 esophoria	Central, unsteady	Central suppression
13	26	+1.50 / −3.75 × 10	−0.25 / −0.75 × 180	0.7	1.2	80	No manifest movement	1 exophoria	Central	No
14	29	−1.00 / −2.00 × 25	−0.75 / −0.25 × 15	0.7	1.0	50	Exophoria	10 exophoria	Central	No
15	31	−5.25 / −3.25 × 170	−5.50	0.9	1.2	40	No manifest movement	Orthophoria	Central, unsteady	No
16	24	+1.00 / −0.25 × 125	+7.00 / −1.25 × 50	1.2	0.5	No	No manifest movement	2 esophoria	Central, unsteady	Central suppression

RE, right eye; LE, left eye;

# *represent subject with no differences between the dominant and non-dominant eye in saccadic latency*.

Seventeen subjects in the control group (CG) had normal or corrected to normal visual acuity (20/20 or better) in each eye and normal binocular vision with good stereopsis (at least 40 arcseconds).

Taking into consideration that two lines of Snellen acuity (BCVA) difference is considered as amblyopia, we classified amblyopia as severe (BCVA worse than 0.2), moderate (BCVA: 0.2–0.5) and mild (BCVA: 0.6–0.9) (Wright et al., 2006; Sapkota et al., 2013).

The experimental protocols were approved by the local ethic committee at Poznan University of Medical Sciences and all experiments were conducted in accordance with the ethical standards laid down in the 1964 Declaration of Helsinki.

### Experiment procedure

**Figure 3:**
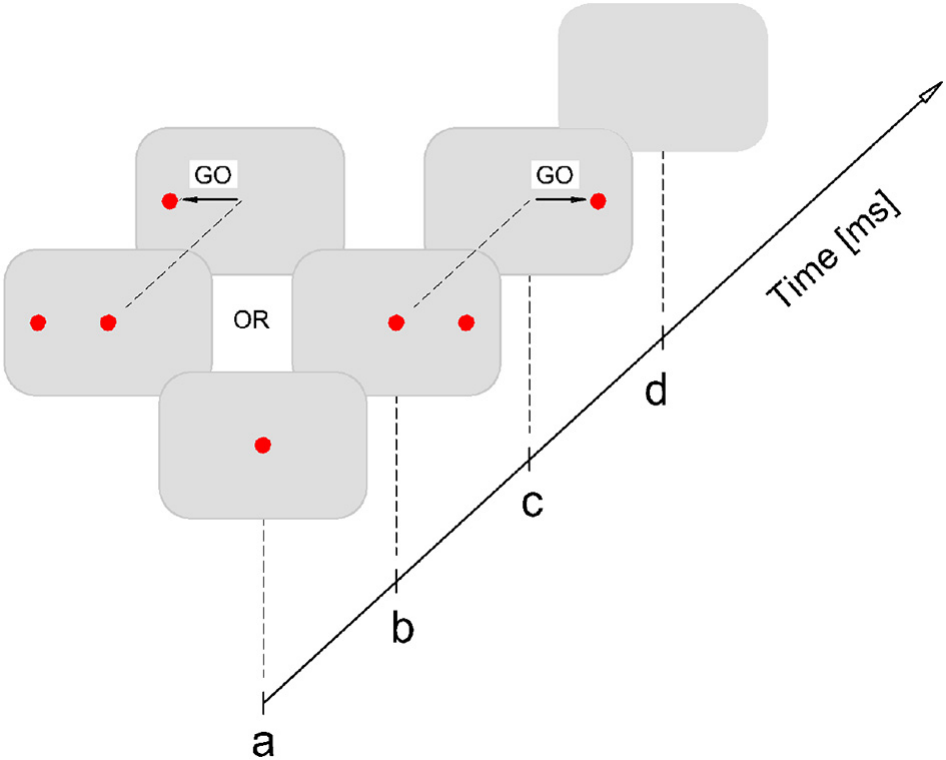
**Experimental procedure**. Participant fixates at central target (a); after 200 ms either left or right peripheral (10°) stimuli is displayed (b); the GO signal for saccadic refixation is the disappearance of the central target, which is randomized in time: 1500–2500 ms (c); after the saccade lands on the peripheral target it stays on for another 200 ms, after that it is switched off and this completes the single stimuli exposure (d). It is followed by the pause of 1000 ms after which the stimulation procedure is repeated again starting from (a).

The subjects were seated in front of the white, uniform wall at the distance of 3 m. The overall room illumination was medium. The visual stimuli for horizontal saccade subtended ~4.5 arcminute. Before the experiment, participants performed the practice trials, consisted with 10 saccades either to the left or right. The experiment consisted of 100 trials for each eye. The experiment was performed under two viewing conditions: monocular amblyopic/non-dominant eye and monocular dominant eye. There was one minute break between trials. The order of viewing conditions was randomized across the subjects, starting either with the dominant or non-dominant eye. The experiment was performed in silence and all subjects have used their optimal refractive correction. In the course of the experiment, the central fixation stimuli (red laser spot) were generated at the beginning, and after 200 ms the peripheral (10°) either left or right stimuli for saccadic refixation response is displayed. The signal for saccadic refixation is given by the disappearance (randomized in time) of central stimuli (GO signal for saccade). Participants were instructed to look at central (fixation) target and when it disappears to make as quickly as possible the saccade toward the peripheral target. The graphical representation of experimental procedure is presented in Figure 3.

### Apparatus

The stimulus, for subject's visual system, is displayed using the miniature laser projectors mounted on the sensor forehead plate. Eye movements (saccadic latencies) were recorded using the Saccadometer Ober Consulting (Ober et al., 2009). The system measures the eye movements using direct infra-red oculography along the horizontal axis (±35°) with high temporal (1 kHz) and spatial (0.1°) resolution. The onset of saccadic response was detected on-line by the measuring system, using 5°/s velocity threshold. The saccadic latencies were measured as the time between the onset of the GO signal (disappearance of the central fixation stimuli) and the onset of saccadic response and stored in Saccadometer memory. They were later transferred to the PC using optical-USB connection, and analyzed there by means of the software provided by the Saccadometer manufacturer.

### Data analysis and statistics

Blinks, error responses (saccades characterized by incorrect direction or amplitude) as well as trials without any responses were rejected from the analysis. Moreover we excluded saccades characterized by latencies smaller than 80 ms and greater than 700 ms. Additionally, the saccades with latencies above and under 2.5 SD were rejected (Van der Stigchel et al., 2010).

For every subject mean, and coefficient of variation (CV) of saccadic latency for dominant eye (DE) and amblyopic eye (AE)/non-dominant eye (NDE) was calculated. Also, we calculated also best fit LATER parameters (σ and μ of the rate of rise) by minimization of the Kolmogorov-Smirnov one-sample test (using SPIC software; Carpenter, 1994).

Data was analyzed using IBM SPSS Statistics. The normality of the gathered data was evaluated using Kolmogorov-Smirnov test. To analyze average values of saccadic latency, its CV and LATER parameters (σ and μ of the rate of rise) we used repeated-measures ANOVA, with one between-subjects factor (group [control subjects and anisometropic subject]) and one within-subjects factors (viewing condition [DE and AE / NDE]). Significant effects of interaction were analyzed further using *post-hoc* Bonferroni-corrected Fisher's LSD test. In the anisometropic group, using Spearman's Rho we calculated correlation between the degree of amblyopia, the mean and variability of saccadic reaction time, as well as σ and μ of the rate of rise for amblyopic eye viewing. For each subject, we also tested an individual differences for DE and AE / NDE (for every subject separately) using SPIC software. Two-sample Kolmogorov-Smirnov was used to compare the observed distributions, while using Student's *t*-test we analyzed individual differences between means of distributions.

## Results

### Mean saccadic latency

**Figure 4:**
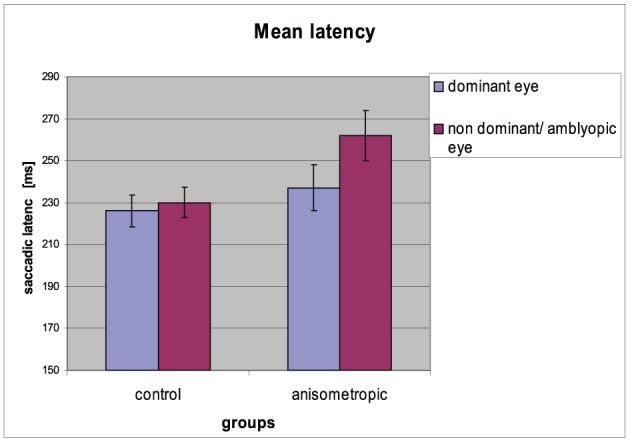
**Mean saccadic latency for controls and anisometropic subjects for particular viewing condition**. Anisometropic subjects performed increased reaction time during amblyopic eye viewing as compared to viewing with the dominant eye. Error bars, ±1 s.e.m.

The mean saccadic reaction times for particular groups in the condition of DE and AE/NDE viewing are presented in Figure 4. The differences in latencies between both eyes were observed. Latency for AE/NDE was longer than for DE [AE/NDE = 246 ± 42 ms vs. DE = 231 ± 38 ms; *F*_(1, 31)_ = 19.586, *p* < 0.001, η^2^ = 0.387], which was proved by significant main effect of *visual condition*.

Results from the statistical tests have shown that in the anisometropic group (AG), the difference in saccade latency between two eyes was increased (262 ± 48 ms vs. 237 ± 45 ms for AE and DE, respectively) compared to the control group (CG) (230 ± 29 ms vs. 226 ± 32 ms for NDE and DE, respectively), which was confirmed by the significant *group* x *viewing condition* interaction [*F*_(1, 31)_ = 10.572, *p* = 0.003, η^2^ = 0.254]. *Post-hoc* Bonferroni test gave a significant result only for the AG (mean difference = 25.000, *p* < 0.001), but not for the CG (mean difference = 3.824, *p* = 0.406). Saccadic reaction times for AE in the anisometropic group was increased as compared to latencies in the NDE in control group, which was revealed by the *post-hoc* test (mean difference = 32,669, *p* = 0.024). Latencies for DE did not differ between the groups (mean difference = 11.493, *p* = 0.397).

### Variability of saccadic latency

**Figure 5:**
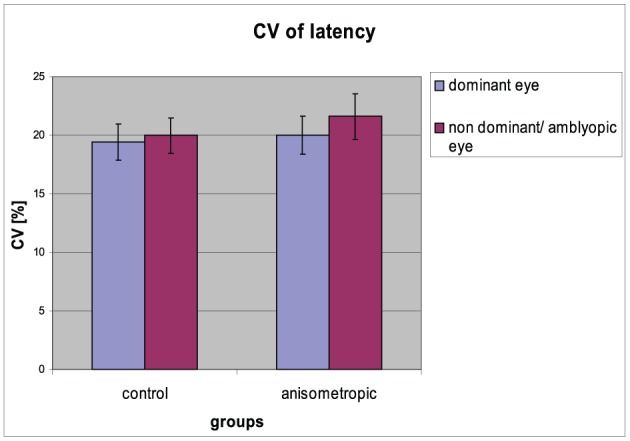
**Coefficient of variation of saccadic latency for controls and anisometropic subjects for particular viewing condition**. There were no significant differences between viewing conditions neither in anisometropic subjects nor in controls. Error bars, ±1 s.e.m.

The CV of reaction time for particular groups in the condition of DE and AE/NDE viewing is presented in Figure 5. In the anisometropic group CV was comparable between DE and AE viewing conditions (AE: 22 ± 8%; DE: 20 ± 7%). The same was in the case of control group (CV for NDE viewing 20 ± 6%; for DE viewing 19 ± 6%), which was also observed in the analysis that has shown insignificant main effect of *visual condition* [*F*_(1, 31)_ = 1,903, *p* = 0.178, η^2^ = 0.058] and insignificant *group* × *viewing condition* interaction [*F*_(1, 31)_ = 0.256, *p* = 0.616, η^2^ = 0.008]. The CV was comparable between the groups, which was proved by the no effect of the group [*F*_(1, 31)_ = 0.601, *p* = 0.444, η^2^ = 0.019].

### Individual analysis

In the experimental group, significant differences were observed in eight subjects in the latency distribution, which was manifested by the significant increase in the mean latency for the AE. Additionally, the increase in the mean latency for AE (without differences in the distributions) was found in three subjects. Only five out of sixteen subjects did not demonstrate any differences between AE and DE. In the control group, significant differences in the latency distribution were observed only in one subject, manifested by the significant increase in the mean latency for NDE. Additionally, the increase in the mean latency for NDE without differences in the distributions were found in four subjects. Twelve out of seventeen controls did not demonstrate any differences between DE and NDE.

### Correlations with the degree of amblyopia

The analysis (using Spearman's Rho) did not reveal any correlations between the degree of amblyopia and the results of mean value and variability in reaction time, as well as σ and μ of the rate of rise for amblyopic eye viewing.

### Later parameters

#### Mean μ of the rate of rise

**Figure 6:**
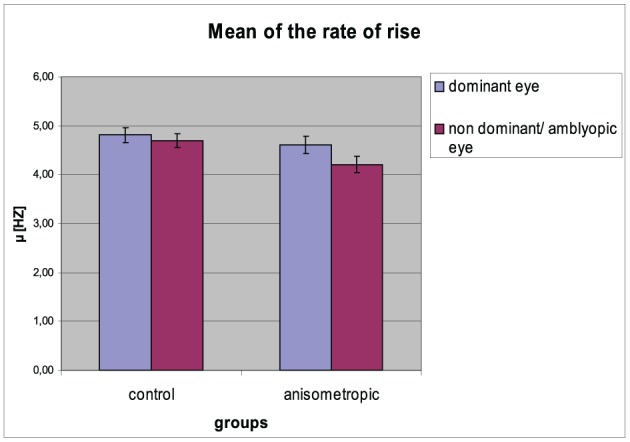
**Mean (μ) of the rate of rise for controls and anisometropic subjects for particular viewing condition**. Anisometropic subjects performed decreased μ during viewing with the amblyopic eye as compared to viewing with the dominant eye. Error bars, ±1 s.e.m.

The mean (μ) of the rate of rise for particular groups in the condition of DE and NDE/AE viewing is presented in Figure 6. The differences in μ of the rate of rise between both eyes were observed. The μ decreased for the NDE / AE, which was confirmed by the main effect of viewing condition [AE/NDE = 4.46 ± 0.67 vs. DE = 4.71 ± 0.67; *F*_(1, 31)_ = 21.720, *p* < 0.001, η^2^ = 0.412]. As can be seen in the anisometropic group, the difference in the μ between both *visual condition* was higher (AE: 4.20 ± 0.67 Hz; DE: 4.60 ± 0.72 Hz) than in the control group (NDE: 4.70 ± 0.61 Hz; *DE*: 4.81 ± 0.64 Hz), which was proved by the significant *group × viewing condition* interaction [*F*_(1, 31)_ = 6.677, *p* = 0.015, η^2^ = 0.117]. *Post-hoc* Bonferroni test revealed significant effect only in the anisometropic group (mean difference = 0.389, *p* < 0.001) but not in the control group (mean difference = 0.114, *p* = 0.146). The μ of the rate of rise for AE in the experimental group decreased when compared to μ for the NDE in the control group which was confirmed by the *post-hoc* analysis (significant only for the AE/NDE condition, mean difference = −0.496, *p* = 0.032). The μ of the rate of rise for DE did not differ between the groups (mean difference = −0.212, *p* = 0.376).

#### Variability (σ) of the rate of rise

**Figure 7:**
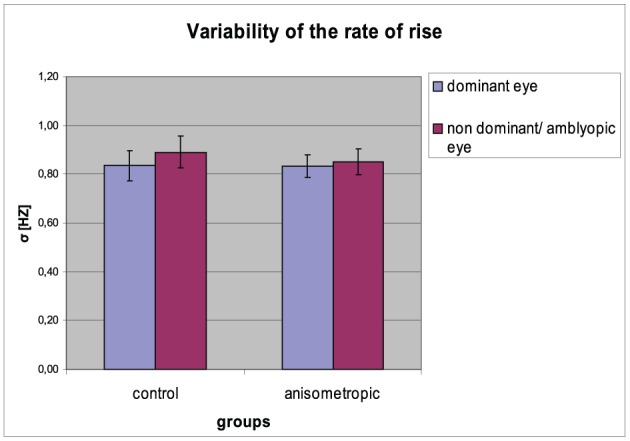
**Variability (σ) of the rate of rise for controls and anisometropic subjects in particular viewing condition**. There were no significant differences between viewing conditions neither in amblyopes nor in controls. Error bars, ±1 s.e.m.

The σ of the rate of rise for particular groups in the condition of DE and NDE viewing is presented in Figure 7. The σ of the rate of rise was comparable between two viewing conditions both in the anisometropic group (for AE: 0.85 ± 0.21 Hz; for DE: 0.83 ± 0.19) and in the control group (for NDE: 0.89 ± 0.27 Hz; for DE: 0.84 ± 0.26 Hz). It was confirmed by the statistical analysis, showing insignificant main effect of viewing condition [*F*_(1, 31)_ = 1.381, error *df* = 31, *p* = 0.249 η^2^ = 0.043] and insignificant *group* × *viewing condition* interaction [*F*_(1, 31)_ = 0.369, error *df* = 31, *p* = 0.548 η^2^ = 0.012). The CV did not differ between the groups, which was proved by the insignificant effect in the group [*F*_(1, 31)_ = 0.075, error *df* = 31, *p* = 0.786 η^2^ = 0.002].

## Discussion

The main finding of this study is the significant difference of saccadic reaction time between the amblyopic and dominant eye in subjects with anisometropic amblyopia. In the control group, saccadic latency for dominant and non-dominant eye was not significantly different. Other authors also reported prolongation of saccadic latency in subjects with amblyopia (see Ciuffreda et al., 1978; Niechwiej-Szwedo et al., 2010). To the best of our knowledge, this is the first study of saccadic latency, carried out using the delayed task in individuals with anisometropic amblyopia. Although delayed saccades are usually applicable when investigating the ability to suppress “automatic” visually triggered saccades (e.g., in Parkinson's disease, Tourette Syndrome or ADHD) (see Munoz et al., 2007), we implement this paradigm in order to engage the central retina in the programming and saccade execution process. However, during preliminary phase of this study, we observed that simultaneous presentation of the central and peripheral target, causes difficulties in the selection of the central point/target during amblyopic eye viewing. For this reason, we decided to use a 200 ms interval between the appearance of the central (as first) and peripheral target in order to indicate which of them is the central. Such a short time interval should not affect the preparatory phase and from the practical point of view appeared very helpful.

Ciuffreda et al. (1978) studied saccadic latencies in 13 subjects (six with constant strabismic amblyopia, three with amblyopia without strabismus and four with intermittent strabismus with or without amblyopia). In the group of constant strabismic amblyopia, the researchers reported an increased latency in three out of six subjects. In the group of amblyopia without strabismus (anisometropic type) they observed increased saccadic latency in two out of three subjects. In the group of intermittent strabismus two out of four subjects had amblyopia and also had longer latency. Unfortunately in Ciuffreda's study (1978) there was no control group. Moreover, there were only three subjects with anisometropic amblyopia and such a small group is not statistically significant. They reported an overall increase of saccadic latency in seven out of eleven amblyopic patients, but they have used different saccadic paradigm, intended for reflexive saccades (fixation target disappears and at the same time appear the peripheral target appears). It is worth to mention, that both in our and Ciuffreda et al. (1978) study not every amblyopic eye showed increased saccadic latency. We cannot exclude the situation that speediness of saccadic decision making is modulated by the compensatory mechanisms (positive and negative outcome of cortical adaptation), what may explain the lack of increase in saccadic latency in some of the amblyopes. Ciuffreda et al. (1978) reported also that after successful amblyopia treatment saccadic latency remained abnormally high. However, they examined only one subject after therapy and future studies should focus on exploring the influence of amblyopia treatment on saccadic latency (we are going to study this topic in the near future).

The study by Niechwiej-Szwedo et al. (2010) included 13 subjects with anisometropic amblyopia and, in contrast to the previous studies, they examined saccadic latency and additionally the saccadic amplitudes and peak velocities during visuomotor task including looking at and pointing with right index finger to a visual target. The saccadic paradigm used by Niechwiej-Szwedo et al. (2010) was also intended for reflexive saccades. Despite of increase saccadic latencies for amblyopic eye (AE: 236 ± 72 ms, DE: 191 ± 38 ms), they reported also an increase in the variability of saccadic latency and no binocular advantage, usually manifested as the reduction of saccadic latency under binocular viewing conditions. Reaching accuracy and reaction time (353 ± 66 ms for patients and 334 ± 86 ms for controls subjects) was comparable between patients and control group but anisometropic amblyopes had significantly longer total mean movement time (Niechwiej-Szwedo et al., 2011). The mean amplitudes and peak velocities were comparable between patients and controls, however they reported greater variability of amplitudes in amblyopic subjects. In the control group, mean saccadic latency was comparable for both right (195 ± 31 ms) and left (192 ± 29 ms) eye (which is in good agreement with our observation) and was significantly shorter for binocular viewing condition (175 ± 32 ms). It is worth to mention that pointing and reaching task requires additional involvement of the dorsal visual stream that has been primarily associated with visually guided reaching and grasping (Hebart and Hesselmann, 2012). To the needs of this study we have assumed that, the releasing of saccadic response is the process taking place exclusively within visual-oculomotor system, and for this reason we decided to compare the findings of saccadic latency measured during visuomotor task with our results. Niechwiej-Szwedo et al. (2010) recorded eye movements binocularly with sampling rate 200 Hz, which results in 5 ms temporal resolution. In our opinion, when we want to analyze differences in saccadic latency in scale of several milliseconds, it is reasonable to use higher sampling rate (at least 300 Hz acc. Juhola et al., 1985; 1000 Hz acc. Ober et al., 2009). Moreover, in the Niechwiej-Szwedo et al. (2010) study there were only 20 saccades for each direction and amplitude, which provided overall 80 saccades per eye. Saccadic latency time constitutes rather a capricious object for the investigator and in order to achieve confidence intervals at the level of ±3 ms we need to acquire at least 100 trials (Ober et al., 2009). Niechwiej-Szwedo et al. (2010) concluded that longer saccadic latency for amblyopic eyes reflects rather the slower afferent (sensory) visual processing than a deficit in the efferent (motor) pathway of the saccadic system. The mean value of saccadic latency reported by Niechwiej-Szwedo et al. (2010) is reduced in comparison to our finding. This is not surprising because it is well known that for the young healthy subject, reflexive saccade latency is significantly faster (typically below 200 ms) than the latency of all forms of voluntary saccade (typically above 200 ms) (Walker et al., 2000; Van Stockum et al., 2011). Both the study by Ciuffreda et al. (1978) and Niechwiej-Szwedo et al. (2010) reported an increase in the variability of saccadic latency during amblyopic eye viewing. This finding was not confirmed by our study. Authors of mentioned studies evaluated variability of latency using its standard deviation (SD). We decided to estimate the coefficient of variation (CV) which, as opposed to SD (that determines absolute differences in characteristics) is a relative measure of the features variability (CV = SD/Mean × 100%). Therefore, the increase of SD may not entail the increase of CV when is accompanied with the adequate growth of mean value (both mentioned studies reported about the simultaneous increase of SD and mean value in the amblyopic viewing condition).

The function of visual pathway from photoreceptors to the visual cortex can be evaluated by means of visual evoked potentials (VEP) recordings. The waveform of VEP contains several characteristic peaks, and in the context of our findings, the most interesting are the peaks C1 (typically 55–70 ms post stimuli onset, generated in primary visual cortex) (Jaśkowski, 2009; Kolb et al., 2013) and P100 (typically 95–110 ms post stimuli onset, generated in dorsal extrastriate cortex of the middle occipital gyrus) (Jaśkowski, 2009; Kolb et al., 2013). Results of VEP responses in amblyopia are rather conflicting and most of the existing studies were focused on the amplitude and latency of the P100. Some authors reported abnormal VEP responses (reduced amplitude and increased P100 latency) in amblyopic subjects (see Sokol, 1983; Parisi et al., 2010) and some did not find any differences between the amblyopic and the sound eye (especially for anisometropic amblyopia) (see Chung et al., 2008; Halfeld Furtado de Mendonca et al., 2013). Parisi et al. (2010) suggested that a delay in postretinal conduction in amblyopia may be responsible for abnormal cortical VEP responses. They reported mean increase ~10 ms (for 25 subjects with anisometropic amblyopia) of the P100 latency. Despite the lack of coherent VEP results identifying the simple neural transportation delay, we cannot exclude the potential influence of such delay on our results.

It is well known, that amblyopia is a cortical deficit, and it is widely accepted view that the primary site of neural deficit in amblyopia is the primary visual cortex (V1), where the information from two eyes is first combined (Hendrickson et al., 1987; Barrett et al., 2004; Roper-Hall, 2007). However, physiological deficits in area V1 are not sufficient to fully explain the whole perceptual deficits observed in amblyopic subjects (Anderson and Swettenham, 2006). Several recent neuroimaging studies indicates that cortical deficits associated with amblyopia are localized within and beyond area V1 (see Anderson and Swettenham, 2006). Hence, it seems to be reasonable to study not only the latency of reflexive saccades but also the latency of voluntary saccades (e.g., delayed saccades) that involves additionally higher cortical processing in the greater extend than the simple saccadic refixation.

**Figure 8:**
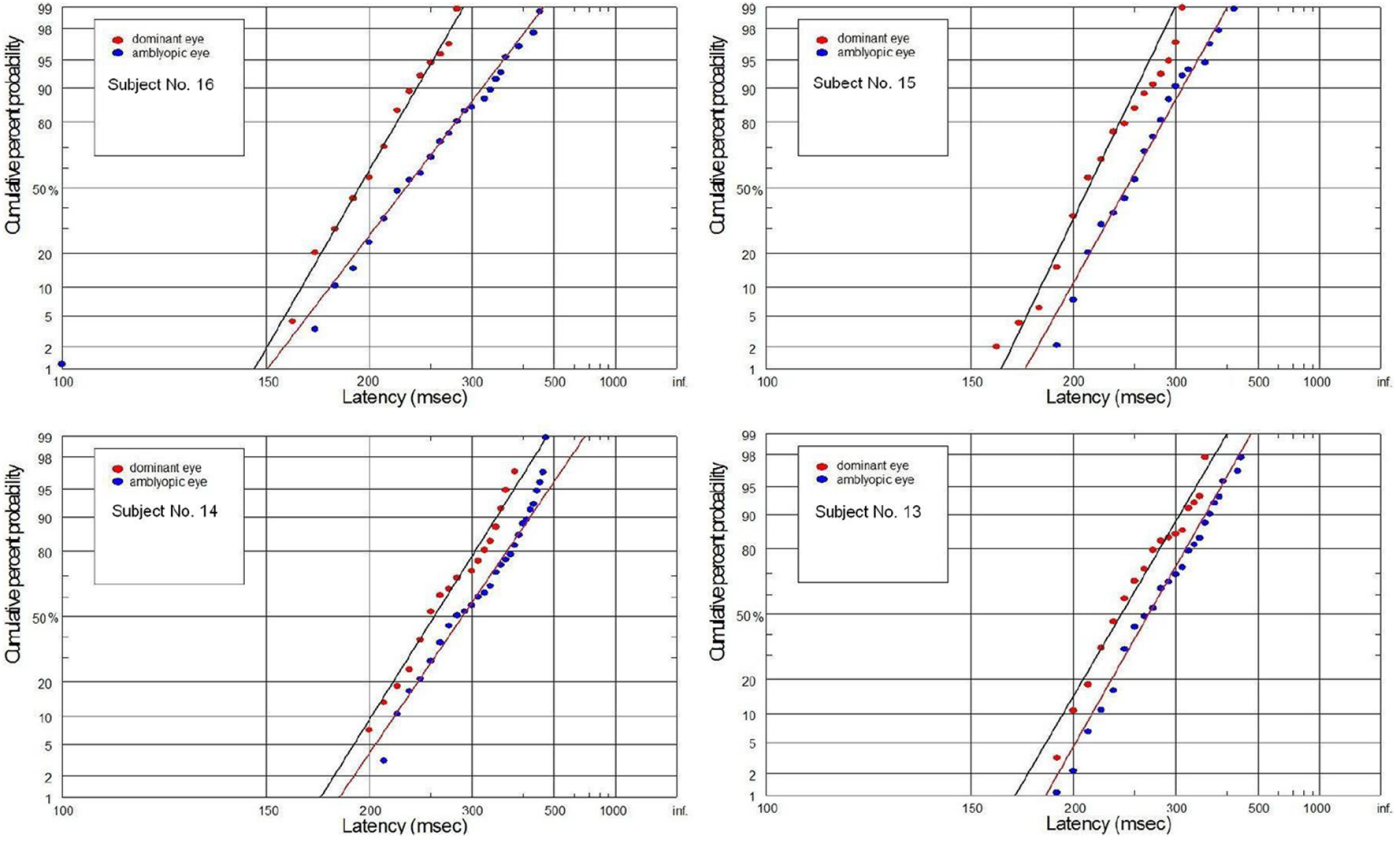
**Latency distribution (plotted as a reciprobits) for selected amblyopes**.

**Figure 9:**
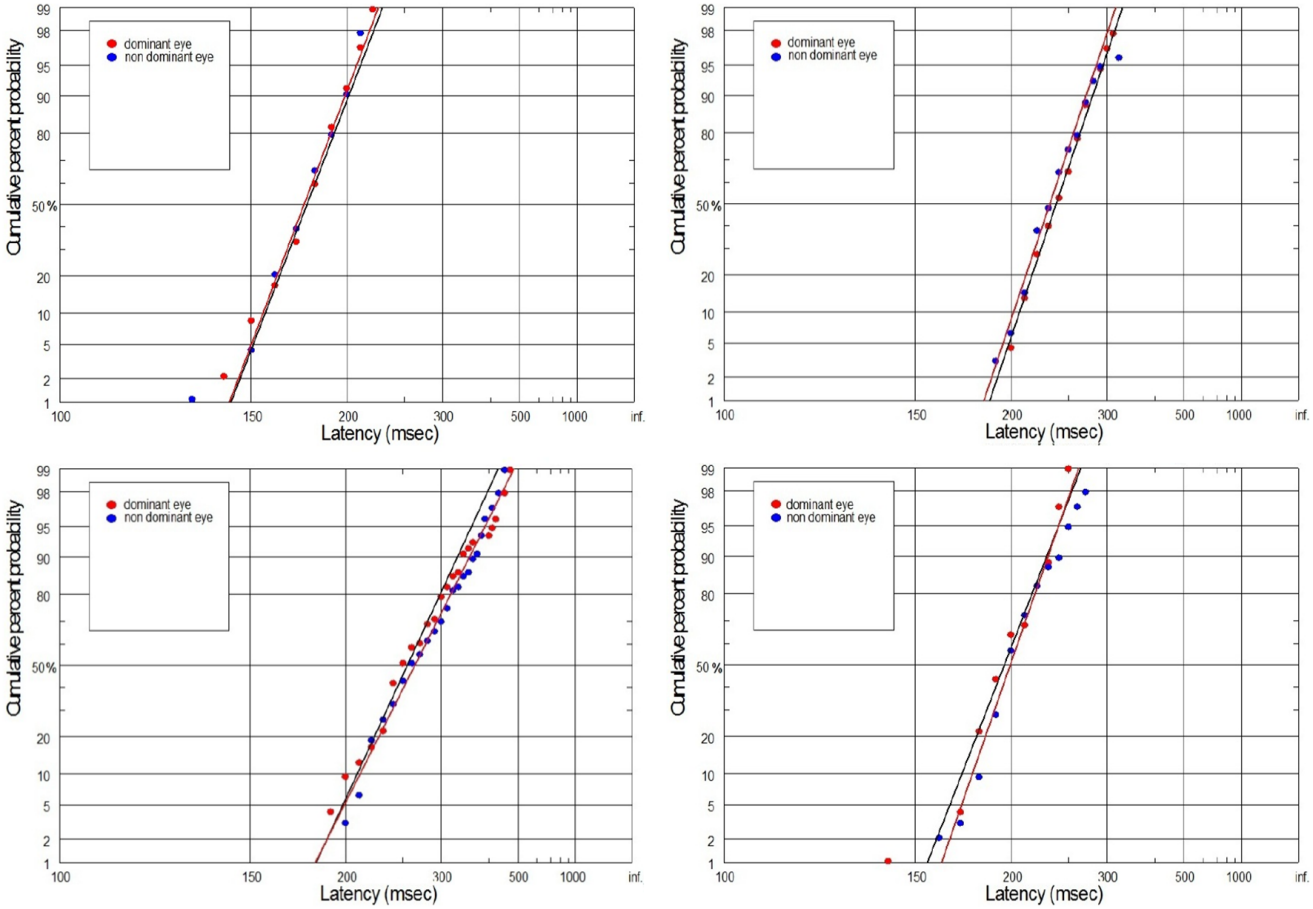
**Latency distribution (plotted as a reciprobits) for selected controls**.

**Figure 10:**
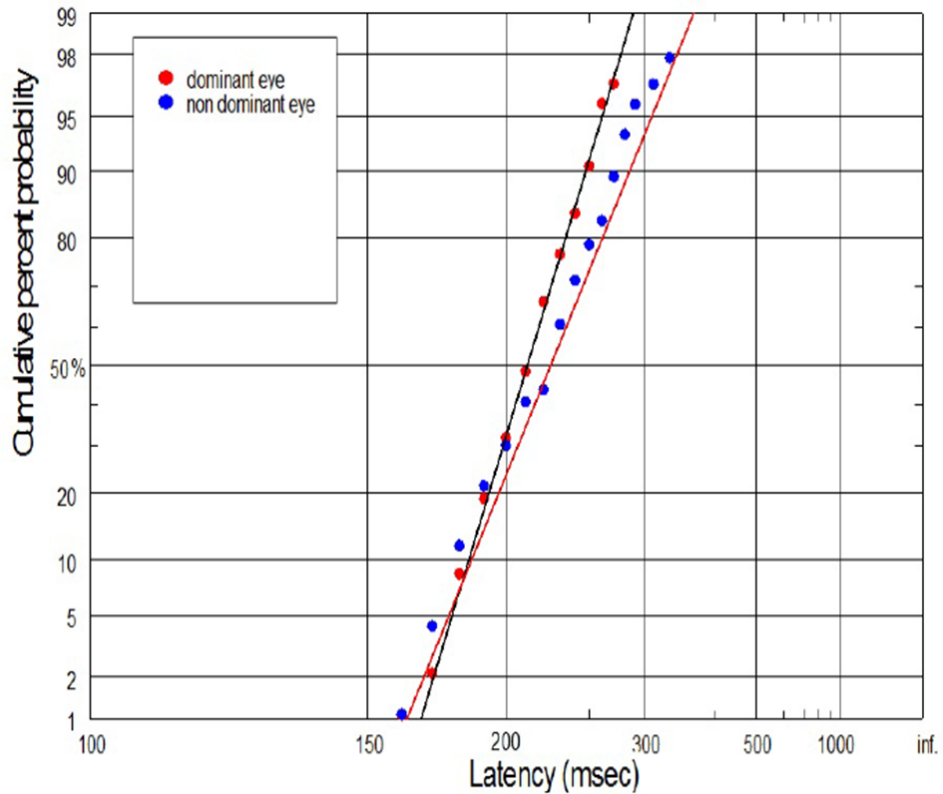
**Example of swiveling and parallel shifting of reciprobit plot for control subject**. In this case we reported significant differences in the latency distribution, manifested by the significant increase of mean latency for NDE.

The supply of information to the visual system can be considered in two dimensions: the speediness and equally important correctness/completeness and both are required for effective saccades programming. For this reason we interpret our finding on the basis of reduced rate of rise of neural activation in the cells involved in saccade initiation for amblyopic eye viewing. As we will discuss in more detail later, the rate of rise of neural activation represents the rate of rise of the decision signal, which (according to the Carpenter's LATER decision model) depends on the rate of information supply to the visual system. We have applied the distributional analysis carried out by means of reciprobit plot, and combined it with the LATER model (Carpenter, 1988; Wardak et al., 2012), in order to plot, compare and interpret the changes in the saccadic reaction time for subjects with anisometropic amblyopia. To the best of our knowledge this the first study that makes such an effort. Neurophysiological evidences supporting the Carpenter's model comes from the studies on rhesus monkeys. Hanes and Schall (1996) studied neural activity of single cells in the FEF, an area that plays a central role in production of voluntary saccades. They reported that neural activity of the cell began to increase ~100 ms before the saccade initiation and peaked shortly after saccade initiation. Saccadic eye movements were initiated only if the neural activity reached a specific and constant threshold level (Hanes and Schall, 1996). The study by Hanes and Schall (1996) has revealed the population of saccadic movement-related neurons, whose activity corresponds closely with the rise-to-threshold of LATER's decision signal (Hanes and Schall, 1996; Reddi et al., 2003). According to the LATER model, the neural signal rises linearly in each trial from an initial level S_O_ to a threshold level S_T_, which initiates the saccade. The rate of rise varies randomly between trials in a Gaussian fashion with mean μ (Reddi et al., 2003). Based on the 100 saccadic responses for each eye, we found significant decrease in mean (μ) rate of rise in anisometropic group (Figure 6) during amblyopic eye viewing, evidenced by the rightward parallel shift of corresponding reciprobit plots (Figure 8). This may be interpreted as a difference in supply of information (decrease in accumulation of visual information) acquired by means of the central retina, that delays the saccade execution decision. In the control group, the mean rate of rise was not significantly different between the eyes (Figure 6), evidenced by the superposition of the corresponding reciprobit plots (Figure 9). Figure 10 presents the subject without any detectable visual problems, however we reported surprisingly significant difference in saccadic latency between the eyes (manifested by the significant increase of mean latency for non-dominant eye). We cannot exclude the situation that this subject was not properly motivated during experimental procedure because when we re-examined this subject several weeks later there were no differences in saccadic latency between the eyes.

**Figure 11:**
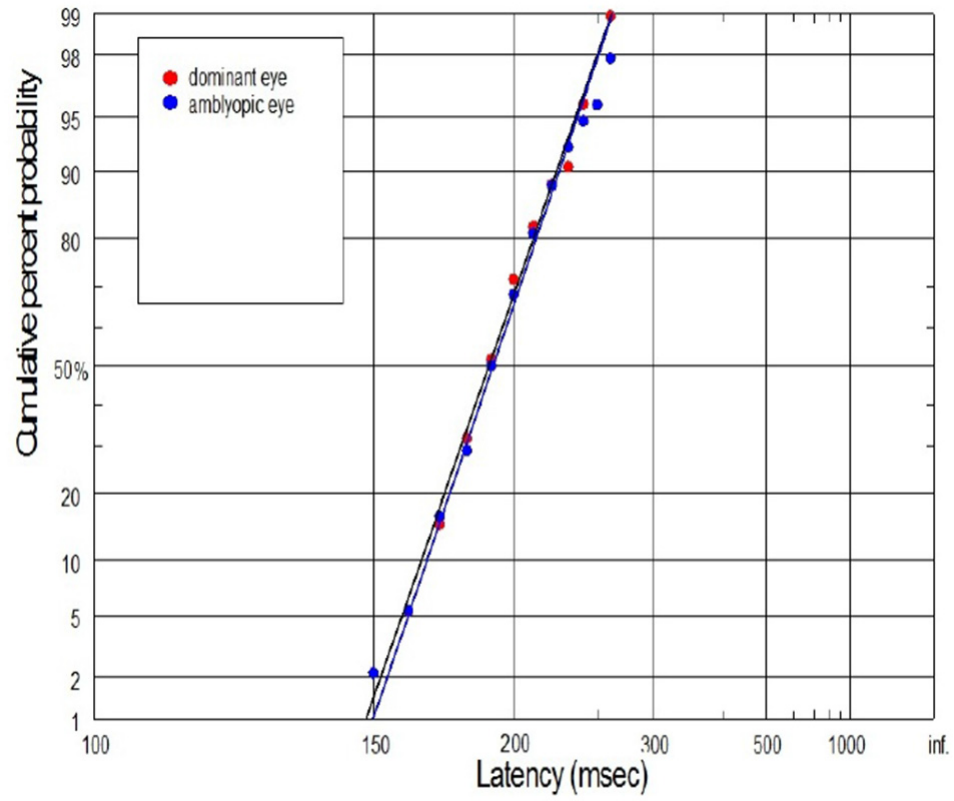
**Example of superposition of reaction time distribution (plotted as a reciprobits) in case of subject (No. 3) with mild anisometropic amblyopia**. There were no difference in saccadic latency between DE and NDE.

We would like also to briefly describe the history of subject No. 15. In this case, the visual acuities of RE and LE were 0.7 and 1.2, respectively, during the subject's first visit, and then a proper refractive correction (including full astigmatism correction of the RE) was prescribed in the secondary school. After several months of regular wearing the new glasses, the visual acuity of the RE improved but still was slightly reduced. Although the experimental test for this subject was performed after treatment, we still decided to join this subject to the experimental group since there are still two lines difference in the Snellen visual acuity between RE and LE. Abnormal VEP response was also recently reported in this subject. In Figure 11, the subject with mild amblyopia (No. 3) in the right eye was presented. In this case, saccadic latency was paradoxically symmetrical in the right and left eye. The individual differences observed in some subjects are quite likely to rise from the compensatory mechanisms at the cortical level (e.g., subject No. 3), or psychophysical higher level factors (e.g., control subject in Figure 10). Furthermore, we should always remember that saccadic latency depends on many factors, including the lowest-level factors such as luminance and contrast of the stimulus and the higher-level factors such as urgency or prior probability. Hence, in our experiment the room illumination and stimulus properties was kept uniform in order to provide repeatable testing conditions.

The increase of saccadic decision-making time (latency) in amblyopia may result from a less efficient (slower) processing of visual information and/or from the poorer quality of information acquired by means of central retina. However, on the basis of our findings we cannot conclusively state which of those two potential mechanisms have a dominant effect on the increase in saccadic latency in our amblyopes. It might be interesting for future studies to explore this topic.

The nature of spatial vision degradation in amblyopia is yet not fully understood. Spatial undersampling (i.e., reduced number of neurons and alternation in the spacing of retinal and cortical receptors) (Levi and Klein, 1996) and uncalibrated neural disarray (i.e., normal number of neurons but lack of calibration in the spatial array of cells covering the visual field) (Field and Hess, 1996) are the two major hypothesis for the losses of spatial vision in amblyopia (Field and Hess, 1996; Levi and Klein, 1996; Wang et al., 1998). However, we cannot decide which of those two proposed explanation have a dominant effect on our result. Both neural undersampling because of a decrease in neuronal spatial sampling density (Wang et al., 1998) and neural disarray (irregular sampling) because of disordered spatial arrangements of cells (Demanins et al., 1999) may reduce the speediness of saccadic decision making time.

Under physiological condition, the attempted steady fixation on a stationary visual target, does not cause the eye to remain motionless. High resolution oculomotor recordings with search coil, allowed to distinguish three types of fixational (involuntary) eye movements: tremor (very fast ~90 Hz oscilations superimposed on drifts), drifts (slow meandering movement that occur between microsaccades) and microsaccades (small, typically less than 0.5° in amplitude involuntary saccades that occur during attempted fixation) (Martinez-Conde and Macknik, 2009; Otero-Millan et al., 2014). See Tables 1–3 from Martinez-Conde et al. (2004) for the detailed characteristics of fixational eye movements. Ciuffreda et al. (1979) studied fixational eye movements in amblyopic subjects. They reported increased drift during monocular viewing with the amblyopic eye. Several authors reported also fixation instability (measured as the dispersion of the eye position during attempted fixation) during amblyopic eye viewing (see Gonzalez et al., 2012; Subramanian et al., 2013). The increase of fixation instability during amblyopic eye viewing may result from increased tolerance of target eccentricity, allowing the observed target to depart further away from the fovea, before calling for the corrective, re-centering saccade. More recently, Shi et al. (2012) found increased amplitude and reduced frequency of microsaccades during monocular viewing with the amblyopic eye in subjects with anisometropic amblyopia. Microsaccade parameters for viewing with the fellow eye were comparable to those in subjects with normal vision. Rolfs et al. (2006) by the use of delayed saccade task have shown that saccadic latency is increased for saccades that occur shortly after microsaccades and is decreased when the microsaccades occurred up to 50ms before the GO signal (target disappearance) for saccades (Rolfs et al., 2006). For that reason the post saccadic refractive periods, which are generated intrinsically by the eye fixation function, can interfere with the externally applied saccadic stimuli being non-time coherent with the intrinsic refractive periods. Assuming that this kind of overlap may appear purely randomly, it should not contribute to the absolute values of the mean latency time but may increase its variability. However, the potential impact of abnormal fixational eye movements in amblyopia on saccadic latency was not confirmed by our results—we did not report any differences in saccadic latency variability between the amblyopic and control group. To the best of our knowledge there is a lack of studies exploring the influence of abnormal fixational eye movements (e.g., increased fixation instability) often observed in amblyopia on the latency of the subsequent saccade. It seems to be interesting for future studies to explore this topic.

Amblyopia is often also associated with temporal instability and spatial distortion (Sireteanu et al., 2008). Although Sireteanu et al. (2008) observed these deficits mainly in strabismic and deep amblyopia, we cannot exclude their effect on our results. This deficits may affect the perception of the disappearance of the central target what may in result delay the saccade initiation in our experiment.

## Conclusions

We found that there is no difference in saccade latency between the right and left eye in the control (without detectable visual deficits) group and significant difference in the anisometropic group. The comparison of saccadic latency between the right and left eye seems to be a useful extension of standard optometric/orthoptic or ophthalmologic examination, especially in subjects with amblyopia. Still, the potential use of distributional analysis of saccadic latencies as a diagnostic tool in amblyopia, should take into account large physiological variability of saccadic latency time and requires the acquisition of at least 100 saccadic responses for each eye. Additionally, it is important to remember that saccadic latency constitutes rather a capricious object for the investigator and depends upon many factors, including the nature of the stimulus, amount of available information, urgency, or prior probability. We hope, that this study contribute to a better understanding of the visual deficits and neural mechanisms underlying them in amblyopia.

### Conflict of interest statement

Witkowska D., Gryncewicz W., Gryncewicz W., and Ober J. declare work for the Ober Consulting company. The coauthors have declared that no competing interests exist. The authors declare that the research was conducted in the absence of any commercial or financial relationships that could be construed as a potential conflict of interest
